# Computed Tomography and Magnetic Resonance Imaging for Congenital Hearing Loss: A Retrospective Study

**DOI:** 10.5152/eurasianjmed.2023.22196

**Published:** 2023-10-01

**Authors:** Ercan Kurt, Mahmut Çoraplı, Cemil Oktay, Abdulkerim Olgun, Mehmet Emin Parlak

**Affiliations:** 1 Department of Otolaryngology, Adıyaman Training and Research Hospital, Adıyaman, Turkey; 2 Department of Radiology, Adıyaman Training and Research Hospital, Adıyaman, Turkey; 3 Department of Plastic and Reconstructive Surgery, Atatürk University Hospital, Erzurum, Turkey; 4 Department of Child Healthy and Diseases, Kahta State Hospital, Adıyaman, Turkey

**Keywords:** Cochlear implant, cochlear nerve, ear MR, deafness

## Abstract

**Objective:**

The aim of this study is to evaluate the relationship between the cochlear nerve and the anatomical structures of the cochlea and internal acoustic canal in patients with congenital hearing loss.

**Materials and Methods:**

Temporal tomography and magnetic resonance images of 44 patients (88 ears) with non-syndromic congenital hearing loss were retrospectively analyzed between 2018 and 2021. Patients were divided into 2 groups according to cochlear nerve hypoplasia. Cochlear nerve canal width, cochlear basal/middle turn widths, and internal auditory canal widths were examined.

**Results:**

Cochlear nerve hypoplasia was detected in 18.2% (n = 16) of the patients and all of the patients with cochlear nerve hypoplasia had severe hearing loss. A statistically significant difference was found between the structures’ widths in patients with and without cochlear nerve hypoplasia, in cochlear nerve canal and coronal width of the internal auditory canal. When stenosis is accepted as <1.4 mm for cochlear nerve canal and <3.80 mm for coronal width of the internal auditory canal, cochlear nerve hypoplasia differs statistically between the groups in measurements (respectively; *P* < .001, *P* = .018).

**Conclusions:**

In patients with sensorineural hearing loss, cochlear nerve hypoplasia may accompany. Anatomical structures are important in predicting cochlear nerve hypoplasia from temporal computed tomography. Cochlear nerve hypoplasia should be suspected if the cochlear nerve canal and coronal width of the internal auditory canal are less than 1.4 mm and 3.8 mm, respectively, on temporal computed tomography.

**Main Points**

- The cochlear nerve canal (CNC) diameter smaller than 1.4 mm in temporal bone computed tomography (CT) in newborn children increases the risk of cochlear nerve (CN) agenesis.
- In temporal bone CT in newborn children, the diameter of the internal acoustic canal smaller than 3.8 mm increases the risk of CN agenesis.
- If the CNC diameter is less than 1.4 mm and the internal auditory canal (IAC) coronal diameter is less than 3.8 mm in the temporal CT of pediatric patients who are candidates for cochlear implants, the CN should be evaluated with magnetic resonance imaging. Cochlear implant is contraindicated in patients with CN agenesis and evaluated for brain stem implant.
- Since the risk of total hearing loss is very high in patients with CN hypoplasia or agenesis, hearing thresholds should be checked with auditory brainstem response.

## Introduction

We use temporal computed tomography (CT) and magnetic resonance imaging (MRI) for inner ear anomalies (cochlear aplasia, cochlear hypoplasia, incomplete partitions, common cavity and complete labyrinthine aplasia-Michel deformity) to see the cochlear nerve (CN) of patients diagnosed with sensorineural hearing loss (SNHL).^1^ While temporal CT gives clear information about mastoid cell aeration, cochlear anomalies, internal acoustic canal, and facial nerve course, temporal MRI gives information about CN and intracranial structures. Most of the SNHL patients are not detected with a cochlear anomaly. With the increase in the quality of imaging methods, clearer findings about the internal acoustic canal (IAC), cochlear nerve canal (CNC) diameter, and CN diameter are obtained.^2^ Many studies reported that there is a relationship between CNC and CN and between SNHL and cochlear hypoplasia (CH).^3-5^ These findings are important for autologists because CNC stenosis can be an important finding in SNHL patients, and absolute knowledge of CN should be obtained by performing a temporal MRI in these patients. In our study, we examined the relationship between hearing levels of SNHL patients and CNC and IAC diameter and CN.

## Materials and Methods

### Participants

Ethical approval of the study was obtained from the Adıyaman University, non-interventional clinical research Ethics Committee (Approval Date: January 18, 2022, Approval No: 2022/1-6). The study is a retrospective study, and temporal CT and MRI of patients aged 1-4 years who were previously diagnosed with total SNHL were evaluated by retrospective archive scanning. The families of the patients were informed about the study during their routine controls and their written consent was obtained. In Adıyaman Training and Research Hospital, 44 patients (88 ears) who were non-syndromic, aged 1-4 years, and diagnosed with bilateral total SNHL were included in the study between 2018 and 2021. Syndromic patients and patients with unilateral hearing loss, meningitis, auricular atresia, otitis media, otitis externa, and hearing loss as a result of trauma were excluded from the study. Fifteen patients were excluded from the study. Diagnosis of hearing loss was made by auditory brainstem response (ABR) test.

### Acquisition and Interpretation of Images

A 64-slice multidetector HRCT (High Resolution Computed Tomography) scanner (Toshiba Aquilion model, Toshiba Medical, Tokyo, Japan) was used for all temporal bone HRCT examinations. All MRI examinations were performed using a 1.5 Tesla MRI device (Achieva 1.5 T, Philips Medical System, Koninklijke, Netherlands). Temporal CT and MRI images were obtained in the supine position. Axial and coronal thin-section images were obtained, which showed the temporal bone structures best and allowed measurements to be made. As described in the study by Yi et al,^6^ CNC, axial images at the CN entrance localization, IAC entrance axial images, IAC length, and mid-point diameter were measured using coronal sections. Basal and middle turns of the cochlea were measured from outside to outside using axial images. The status of CN (normal/hypoplasia/absent) was assessed using thin-section MRI images. Measurements were performed using the institutional database system (Oracle database V1.10.43.134). Measurement and analysis of all parameters were done by manually enlarging the images with the consensus of 2 radiologists with 11 and 8 years of experience in the field (M.Ç. and C.O.). Both radiologists made measurements without knowing the hearing status of the patients.

### Audiologic Evaluation

Auditory brainstem behavior (ABR) was performed on all patients younger than 6 years of age with suspected hearing loss in our clinic and their hearing levels were checked. Auditory brainstem behavior was performed under general anesthesia by a single audiologist. Minimum hearing levels by were determined adding 5 dB when patients did not respond at their maximum hearing level. Hearing loss ratings were categorized as very mild (16-40 dB), mild (41-55 dB), moderate (56-70 dB), severe (71-90 dB), and very severe (total) (91 db and above).

### Statistical Analysis

Statistical analysis was performed to evaluate the relationship between the anatomical structures of the cochlea and IAC with CN hypoplasia. Descriptive statistics were summarized with means and standard deviation (minimum-maximum) for continuous variables, and numbers (n) and percentages for categorical variables. The normal distribution of the results was checked by the Kolmogorov–Smirnov test for continuous variables, and according to normality test, the results were analyzed using the independent samples *t*-test or Mann–Whitney *U* test. The cutoff for CNC and coronal width of the internal auditory canal (CW-IAC) stenosis were accepted as the 25th percentile, and the relationship with CN hypoplasia was evaluated with Fisher’s exact tests and chi-square test. Spearman correlation analysis was performed to evaluate the CNC measurements and ABR results. All statistical analyses were carried out using the Statistical Package for Social Sciences software, version 23.0 (IBM SPSS Corp.; Armonk, NY, USA), and significance level was considered *P* < .05.

## Results

In this study, 44 patients and 88 ears were included, and 52.2% (n = 23) of the patients were female and 47.8% (n = 21) were male. The average age of the patients is 20 months (min: 12 months, max: 48 months). In ABR, 18.2% (n = 16) of 88 ears had very mild (16-40 dB), 10.2% (n = 9) had mild (41-55 dB), 10.2% (n = 9) had moderate (56-70 dB), 11.4% (n = 10) had severe (71-90 dB), and 50% (n = 44) had very severe (91 db and above) hearing loss. Cochlear malformations (n = 1 Michel, n = 2 cochlear aplasia, n = 2 incomplete partition type I) were detected in 5 ears in temporal CT, and CN hypoplasia was detected in 16 ears in temporal MRI. All patients with CN hypoplasia (n = 16) had very advanced SNHL. The temporal CT measurement data of patients with and without CN hypoplasia are given in Table 1.

In our patients with CN hypoplasia, the CNC width was found to be statistically significantly lower (*P* < .001, *t* = 8.095). The median value of the CNC width was measured as 1.90 mm (interquartile range 1.40-2.20) for all patients, and when the 25th percentile (<1.4 mm) cutoff was accepted for CNC stenosis, CNC measurement and CN hypoplasia differed statistically significantly (*P *< .001, χ^2^ = 56.168). Data on the dispersion of CN hypoplasia in imperfections with CNC stenosis (<1.4 mm) are shown in Table 2.

Cutting value of CNC width <1.4 mm; 94% (95% CI 0.68-0.99) sensitivity, 93% (95% CI 0.84-0.97) specificity, 75% (95% CI 0.50-0.90) positive predictive value, and 98% (95% 0.90-0.99) negative CN hypoplasia predicts predictive value. A statistically significant but negative and weak correlation was found in the correlation analysis between CNC measurement and ABR values of the patients (*r* = −0.365, *P* < .001).

In this study, for those with CN hypoplasia, CW-IAC width was found to be statistically significantly low (*P* = .009, *z* = −2.630). The median CW-IAC value was measured as 3.90 mm (interquartile range 3.80-4.20) for all patients, and when the 25th percentile (<3.80 mm) cutoff is accepted for CW-IAC stenosis, W-IAC measurement and CN hypoplasia are statistically differentiable (*P* = .018, χ^2^ = 6.818).

The results of the distribution of CN hypoplasia in this case with CW-IAC stenosis (<3.80 mm) are shown in Table 2. Statistical data for predicting CN hypoplasia of <3.80 mm cutoff value of CW-IAC width are shown in Table 3.

In other measurements of the IAC and cochlea (basal turn, middle turn), there was no statistically significant difference in patients with or without CN hypoplasia (Table 1).

## Discussion

There are many causes of SNHL in children. Cochlear anomalies, IAC anomalies, and CN anomalies are some of them. Imaging methods are important in children with hearing loss and temporal CT is usually applied first. In addition, imaging methods give us information about the etiology of SNHL. For example, in patients with normal CNC but CN hypoplasia/aplasia, the cause of hearing loss may be interpreted as not congenital but acquired SNHL (vascular, traumatic, compression, or inflammatory). Temporal CT is used to see the labyrinth and cochlear structures of patients diagnosed with SNHL, and MRI is used to see the vestibulocochlear nerve and facial nerve. Recent studies have found that 10% of newly diagnosed SNHL pediatric patients have CN anomaly.^6^ Cochlear nerve anomaly can be together with cochlear anomalies or alone. Sometimes labyrinth anomalies may accompany. Although IAC is normal in some patients diagnosed with SNHL, CN hypoplasia or agenesis may be present.^7^ Inner ear malformation rate in patients with SNHL varies between 20% and 30%.^8^ In the study by Miyasaka et al,^9^ CN hypoplasia or aplasia was found on MRI in 19% of SNHL patients.^9^ In our study; MRI showed CN anomaly in 16 of 88 ears (18%), and cochlear anomaly in 5 ears on temporal CT (1 Michel, 2 cochlear aplasia, 2 incomplete partition type I). Congenital and acquired damage in children with SNHL may cause CN anomalies. In some studies, it has been stated that IAC stenosis may be suggestive of CN hypoplasia or aplasia in temporal CT.^10^ In this patient group, clear information about CN should be obtained by temporal MRI. There may be CN hypoplasia or aplasia without IAC stenosis. This condition may be the result of degeneration of nerve fibers due to vascular, traumatic, compression, or inflammatory causes.

In the study by Komatsubara et al^5^ on temporal CT, when patients with CNC stenosis were examined by temporal MRI, CN hypoplasia was found with 88.9% sensitivity and 88.9% specificity^5^. Similar results were obtained in our study with 94% sensitivity and 93% specificity.

In the study by Yi et al^6^ of patients with unilateral SNHL, most of the ears (57%) with unilateral SNHL had CNC stenosis/atresia. If the diameter of the CNC was less than 1.4 mm, pure tone auditory averages were more than 70 dB HL in most ears.^6^ In our study, all patients with CN hypoplasia had advanced SNHL.

Yan et al^11^ in their retrospective study of patients with CN aplasia/hypoplasia found that hypoplastic CNC may be more of an indicator of CN aplasia/hypoplasia than a narrow IAC. In our study, most patients with CN hypoplasia in SNHL had CNC stenosis, and IAC stenosis was rare (Table 1).

Tahir et al^12^ in their study on SNHL patients with CNC stenosis, CN was found to be hypoplastic or aplastic when the CNC diameter was less than 1.5 mm and the IAC diameter was less than 2 mm.^12^ In our study, CN hypoplasia was found mostly (75%) when the CNC diameter was less than 1.4 mm.

It is important to have clear information about the condition of the cochlea, IAC, CNC, and CN in order to plan the treatment in SNHL patients. It should be known that SNHL patients may have CNC and/or IAC stenosis, even if the patient’s cochlea is normal in temporal CT. Cochlear nerve hypoplasia should be suspected when patients with SNHL have a CNC diameter of less than 1.4 mm on temporal CT and a CW-IAC of less than 3.8 mm. Cochlear nerve canal stenosis is more significant than IAC stenosis. If CN hypoplasia is suspected, clear information about CN must be obtained with MRI. We think that this information will be a guide for otolaryngologist specialists who are interested in cochlear implants. Temporal CT should be examined in detail in patients who will undergo cochlear implants. In patients with a coronal diameter of the inner acoustic canal less than 3.8 mm, further audiological testing such as cortical hearing test should be performed, and information about the CN should be obtained by examining thin-section temporal MR in detail.

### Limitations

1. The lack of cochlear aplasia patients and the low rate of inner ear anomalies in our study are the factors limiting our study. Multicenter studies with more patients may yield different results.
2. The study’s major limitation is its retrospective design.

## Figures and Tables

**Table 1.** Measurements of the Anatomical Structures of the Cochlear and Internal Acoustic Canal

	Normal CN (n = 72)	CN Hypoplasia (n = 16)	*P*
Cochlear nerve canal	1.97 ± 0.42 (1.2-2.9)	1.08 ± 0.22 (0.8-1.7)	***.001, t = 8.095***
Cochlear basal turn width	5.24 ± 0.52 (4.10-5.90)	5.08 ± 0.54 (4.20-5.60)	.385, z = −0.869
Cochlear middle turn width	3.43 ± 0.39 (2.60-3.90)	3.30 ± 0.4 (2.80-3.80)	.456, z = −0.746
The internal auditory canal width at the porus level	5.88 ± 0.75 (4.10-7.30)	5.82 ± 0.62 (4.90-6.90)	.535, z = −0.620
Coronal width of the internal auditory canal	4.17 ± 0.58 (3.40-6.50)	3.86 ± 0.30 (3.40-4.70)	. ***009, z = −2.630***
Transverse width of the internal auditory canal	7.63 ± 0.89 (5.10-8.50)	7.27 ± 1.29 (5.20-8.50)	.551, *z* = −0.596

CN, cochlear nerve.

**Table 2.** Cutoff Values Determined for Cochlear Nerve Canal and Internal Acoustic Canal Stenosis and Distribution of CN Hypoplasia in These Patients

Variables	Cutoff Values	Normal CN (n = 72)	CN Hypoplasia (n = 16)	Total	*P*
Cochlear nerve canal width	<1.4 mm	5 (25%)	15 (75%)	20 (100%)	***<.001***
	≥1.4 mm	67 (93.5%)	1(1.5%)	68 (100%)	
Coronal width of the internal auditory canal	<3.80 mm	8 (57.1%)	6 (42.9%)	14 (100%)	.018
	≥3.80 mm	64 (86.5%)	10 (13.5%)	74 (100%)	

CN, cranial nerve.

**Table 3.** Prediction Rates for CN Hypoplasia for Cutoff Values Determined for Cochlear Nerve Canal Width and Internal Acoustic Canal Stenosis

Variables	Cutoff Values	Sensitivity (95% CI)	Specificity (95% CI)	Positive Predictive Value (95% CI)	Negative Predictive Value (95% CI)
Cochlear nerve canal width	<1.4 mm	94% (68-99)	93% (83-97)	75% (50-90)	98% (91-99)
	≥1.4 mm				
Coronal width of the internal auditory canal	<3.80 mm	38% (16-64)	89% (78-94)	42% (19-70)	86% (76-92)
	≥3.80 mm				

CN, cranial nerve.
